# Sensitive and reliable detection of *KIT* p.D816V mutation in decalcified archival bone marrow trephines

**DOI:** 10.1007/s00428-024-03973-8

**Published:** 2024-11-14

**Authors:** Miriam Odensass, Stephan Bartels, Jerome Schlue, Guntram Büsche, Hans H. Kreipe, Ulrich Lehmann

**Affiliations:** Institute of Pathology, Hannover Medical School, Carl-Neuberg-Str. 1, 30625 Hannover, Germany

**Keywords:** *KIT* p.D816V, Systemic mastocytosis, Bone marrow trephines, MRNA

## Abstract

The majority of mastocytosis cases are characterized by an activating mutation in the *KIT* gene in codon 816. The detection of this alteration is of importance for proper diagnostic workup. Therefore, reliable and sensitive methods for the detection of *KIT* Codon 816 hotspot mutations in various types of patient samples are required. Since mutated cancer genes are often overexpressed, we evaluated the feasibility and sensitivity of *KIT* p.D816V detection by analysing mRNA/cDNA instead of genomic DNA. From 80 bone marrow trephines harboring a *KIT* p.D816 mutation, seven were only mutated by mRNA/cDNA pyrosequencing and 11 only by digital PCR analysis of genomic DNA. These results clearly demonstrate that detection of clinically relevant mutations in mRNA extracted from routinely processed decalcified archival bone marrow trephines is not only possible in a reliable fashion but under many circumstances advantageous. This enables the direct correlation of genomic data with high-quality morphological evaluation.

**Supplementary information:**

The online version contains supplementary material available at 10.1007/s00428-024-03973-8.

## Introduction

Systemic mastocytosis is a rare but sometimes life-threatening heterogeneous group of diseases characterized by a monoclonal atypical proliferation of mast cells accumulating in one or more organs according to WHO classification [1]. In the majority of cases, it is characterized by an activating mutation in the stem cell factor receptor gene *KIT* in codon 816.

**Fig. 1:**
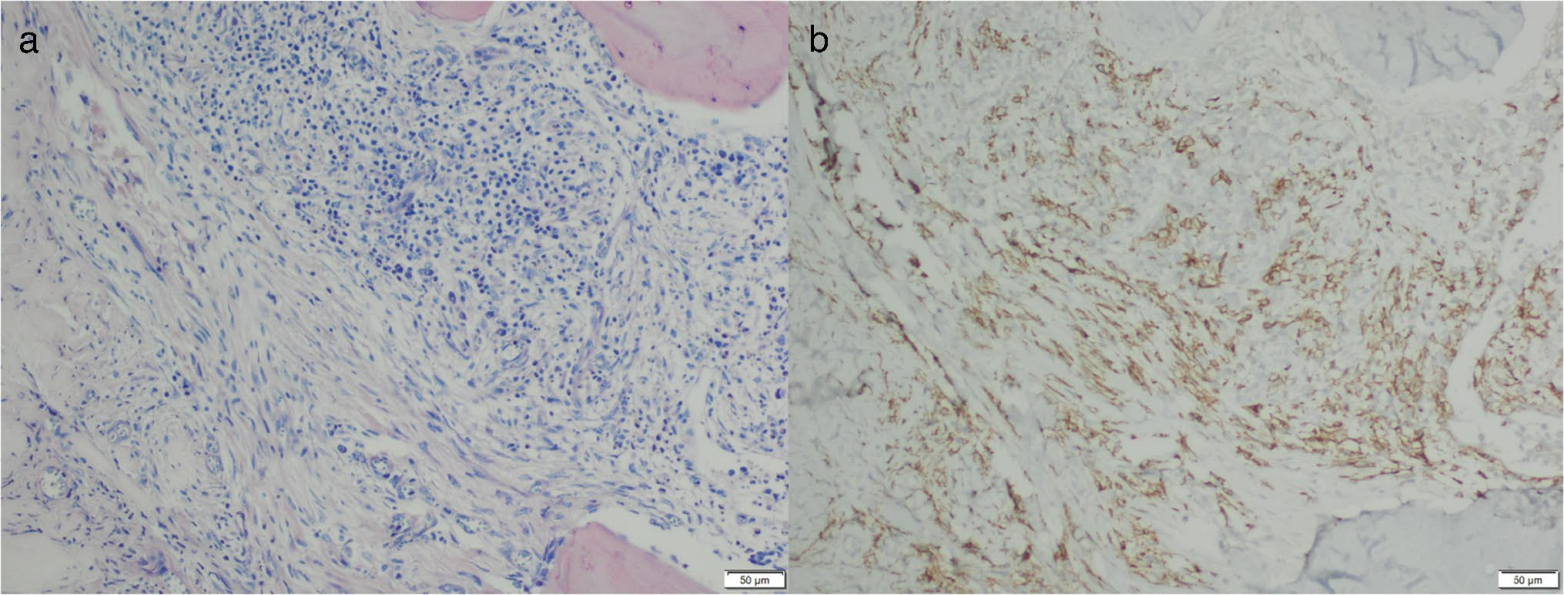
Photos of bone marrow biopsy with a compact mast cell infiltrate mostly consisting of spindle shaped mast cells: **a** Giemsa (100 × magnification) and **b** CD117 immunohistochemistry (100 × magnification)

Since systemic mastocytosis can be an incidental finding in the context of hematological diagnostics, detection of *KIT* Codon 816 hotspot mutations should be possible from all kinds of patient samples sent to a haematological laboratory, including formalin-fixed, decalcified, and paraffin-embedded bone marrow trephines. And the detection method has to be of high sensitivity because, diagnostically, relevant mast cell infiltrates can be quite small. The biopsy plays an essential role in the diagnostics of systemic mastocytosis, because the major diagnostic criterion in the diagnosis of systemic mastocytoses is the detection of compact mast cell infiltrates in the context of extracutaneous tissues (see Fig. 1) [1].

Under many circumstances, mutated cancer-driving genes are also overexpressed. Therefore, we reasoned whether detection of *KIT* Codon 816 hotspot mutation at the mRNA level might be more sensitive if only very small mast cell infiltrates are discernible. In order to test this hypothesis, we compared a standard pyrosequencing assay based on genomic DNA with a pyrosequencing assay based on mRNA/cDNA and a sensitive digital PCR assay targeting genomic DNA.

Greiner et al. [2] could convincingly demonstrate that the allelic burden of *KIT* p.D816V in bone marrow tissue samples is significantly higher compared to peripheral blood or bone marrow aspirate and represents a new biomarker with prognostic significance. For this reason and because of the possibility to correlate morphological and immunohistochemical findings directly with the mutation status, this study focused exclusively on the analysis of DNA and RNA extracted from formalin-fixed, decalcified, and paraffin-embedded bone marrow trephines.

## Materials and methods

### Patient samples

A total of 101 archived biopsies from 101 adult patients, collected between 2017 and 2023, were analyzed including cases with systemic mastocytosis (*n* = 76) and cases in which the WHO criteria for diagnosis of a systemic mastocytosis were not fulfilled (*n* = 25) (Table 1), including fifteen biopsies with inconspicuous mast cells without atypia that were used as negative controls for the definition of the detection limit.

**Table 1:** Main characteristics of the patient cohort

Cohort	Total sum	101 (100%)
Sex	Men	58 (58.3%)
	Women	42 (41.7%)
Diagnosis*	Systemic mastocytosis (SM)	32 (31.7%)
	Systemic mastocytosis with associated hematologic neoplasia (SM-AHN)	42 (41.6%)
	Mast cell leukemia (MCL)	2 (1.9%)
	Criteria not fulfilled	25 (24.8%)
Age	Median	63 years
	Range	20–83 years

^*^76 patient samples fulfilled the criteria for diagnosis of a systemic mastocytosis according to the WHO classification. Among them there were two cases without a *KIT* mutation in the three assays. In six patient samples we detected a *KIT* mutation (c.2447A > T or c.2446G > C) in one or more assay without fulfilling the other criteria for a systemic mastocytosis

Ninety-nine samples were fixed, decalcified, and paraffin-embedded bone marrow trephines, and two were FFPE samples from colon. For details of the patient cohort and tissue processing, see Supplemental Table S1 and Materials and Methods Supplement.

This study was approved by the Hanover Medical Ethics Committee (10977_BO_K_2023).

### DNA/RNA extraction

DNA and RNA extraction and nucleic acid quantification was performed as described [3]. For further details see, Supplementary information.

### cDNA synthesis

For cDNA synthesis the “High-Capacity cDNA Reverse Transcriptase-KIT” (Thermo Fisher Scientific, Waltham, MA, USA) was used following the manufacturer’s instructions, starting with 200–1000 ng of total RNA. For further details, see Supplementary information.

### Digital PCR

Digital PCR experiments were performed by the QuantStudio 3D Digital PCR System platform using the QuantStudio 3D Digital PCR Master Mix V2 (Thermofisher Scientific, Waltham, MA, USA) according to the manufacturer’s instructions. The target sequence was analyzed in duplicate using 65 ng gDNA per replicate. The assay mix “KIT_1314” (Thermofisher Scientific) was used containing a FAM-labelled probe targeting the point mutation c.2447A > T in Exon 17 in the *KIT* gene and a VIC-labeled probe targeting the wildtype allele at position c.2447. For further details, see Supplementary information.

### Pyrosequencing

Pyrosequencing for the analysis of Codon 816 in the *KIT* gene was performed essentially as described [4]. For further details, see Supplementary information.

### Plasmid and oligonucleotide

The technical sensitivities were calculated by quantifying standard dilutions of oligonucleotide containing the *KIT* mutation p.D816V, spanning the intron and the exon 17 of the *KIT* gene and a plasmid containing a cDNA insert with the *KIT* mutation c.2447A > T:p.D816V.

## Results

All 101 routinely processed bone marrow trephines (formalin-fixed, decalcified, and paraffin-embedded) provided both DNA and RNA of sufficient quality for successful genotyping of the *KIT* gene at Codon 816 (100% success rate). Using an oligonucleotide or plasmid as precisely quantifiable template all three assays were able to detect under optimal conditions a single molecule in the reaction mixture (Supplemental Figures S1, S2, and S3).

Eighty samples were positive for a typical activating *KIT* exon 17 hotspot mutation in Codon 816 (p.D816V or p.D816H, for details see Supplemental Table S1). No clear (statistical significant) difference was discernible between dPCR analysis of gDNA and pyrosequencing of cDNA: eleven cases were positive only in the dPCR assay and seven cases were positive only in the pyrosequencing assay using cDNA (*p* = 0.35, McNemar test). In contrast to these results, pyrosequencing of genomic DNA (gDNA) was much less sensitive: In 44 samples, which were tested positive by dPCR of gDNA and/or pyrosequencing of cDNA, no mutation in codon 816 could be found by pyrosequencing using gDNA (false negative), indicating a clearly inferior sensitivity of this approach.

**Fig. 2:**
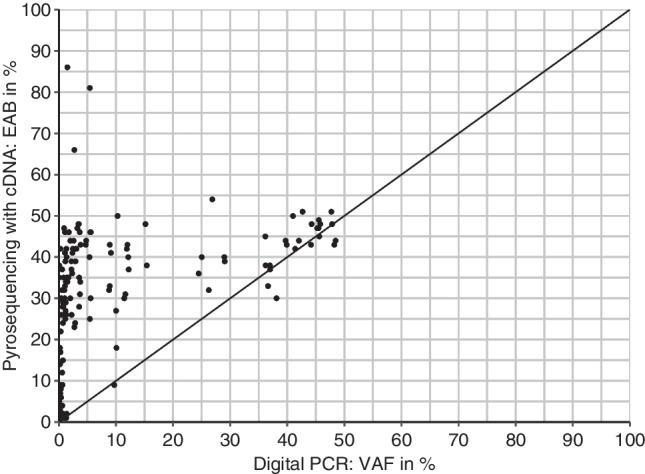
Expressed allelic burden (EAB), measured by pyrosequencing using cDNA as a template, versus variant allele fraction (VAF), measured by digital PCR using genomic DNA as a template

Comparing the variant allele frequency (VAF) determined using gDNA as template with the expressed allelic burden (EAB, following the terminology of Erben et al. [5]) and that determined using mRNA/cDNA as template, it becomes obvious that the latter is much higher especially in cases with low VAF (see Fig. 2). This indicates that under some circumstances, the mutation detection is more sensitive if mRNA/cDNA is used as template.

For a subset of samples (*n* = 25), mutational analyses employing next-generation sequencing (targeted analysis using amplicon-based enrichment) were available for comparison. In five samples negative by NGS *KIT*, a hotspot mutation (p.D816V or p.D816H) could be detected by digital PCR using gDNA as template and/or pyrosequencing of cDNA.

## Discussion

In our study, the success rate for DNA and RNA analysis of routinely processed bone marrow trephines was 100%. In contrast to these results, Agarwal et al. reported a success rate of only 69% for extraction and amplification of gDNA (63 out of 202 bone marrow trephines failed [6], indicating suboptimal fixation and decalcification conditions and/or inefficient DNA extraction. Greiner et al. [2] reported a success rate similar to our study for the analysis of gDNA extracted form 211 FFPE bone marrow trephines. In a much smaller group of 20 bone marrow trephines, Tan et al. achieved also a success rate of 100% for the analysis of gDNA [7]. The number of analyzed archival bone marrow trephines in several previous studies is too small in order to draw any meaningful conclusions about feasibility, reliability, and sensitivity (Kähler et al. 2007: *n* = 5 [8]; Schumacher et al. 2008; *n* = 13 [9]; Kristensen et al. 2011: *n* = 1 [10]).

Sotlar et al. [11] described already in 2003 a real-time PCR assay for the detection of KIT p.D816V in archival FFPE biopsies. However, in contrast to our study, Sotlar et al. analyzed cutaneous manifestations of mastocytosis. Formalin-fixed, decalcified, and embedded bone marrow trephines were only included as negative controls which were all KIT Codon 816 wildtype. Therefore, nothing can be deduced from this data set about the reliability and sensitivity of KIT p.D816V mutation detection in decalcified bone marrow trephines.

Corless et al. [12] reported already in 2006 on the analysis of bone marrow biopsies for the detection of *KIT* codon 816 mutations in gDNA. However, from “Materials and Methods” and the results in table 2 in reference [12], it remains unclear whether the eight bone marrow samples designated as either “formalin-fixed” or “embedded” have been decalcified.

Kristensen et al. [13] performed a direct comparison of gDNA-based versus mRNA-based analysis of *KIT* p.D816V mutation before. However, they did not analyze formalin-fixed specimens. Instead, they compared the results for peripheral blood and bone marrow aspirate, respectively, describing a significant difference in diagnostic yield for gDNA versus mRNA in peripheral blood. The same group also demonstrated analysing various cell line mixtures and fresh patient samples that the potentially very sensitive next-generation sequencing methodology can be false negative if the VAF of the *KIT* p.D816V mutation is very small [14], an observation confirmed by our results analyzing fixed and decalcified bone marrow trephines.

In comparison digital PCR with gDNA which can only detect *KIT* mutation c.2447A > T, cDNA-based pyrosequencing can detect more than one activating *KIT* mutation. This limitation explains some discrepancies in the results especially concerning cases without *KIT* mutation in the digital PCR assay and *KIT* mutation c.2446G > C in the pyrosequencing (ID 42, 69, 70, and 77). Eleven patient samples showed the *KIT* mutation c.2447A > T only in the digital PCR assay (ID 41, 46, 47, 52, 53, 61, 62, 65, 80, 82, and 83) and three patient samples (ID 57, 68, and 96) showed a *KIT* mutation c.2447A > T only in the pyrosequencing assay with cDNA. In addition to a possible inhomogeneous distribution of neoplastic mast cells in the sections for the DNA and RNA extraction, the determination of threshold values was based on an absolute quantification of FAM labelled signals in the digitalPCR (five positive FAM-signals) whereas the results of the pyrosequencing were only based on relative quantification (4% A > T at position 2447 in the forward assay as cut off).

In conclusion, this study demonstrates that reliable and sensitive analyses of nucleic acids extracted from formalin-fixed, EDTA decalcified, and paraffin-embedded bone marrow trephines is possible under routine conditions and that the analysis of mRNA for mutation detection is a useful complement for the comprehensive diagnostic work-up in hematopathology.

In addition, this approach enables the direct correlation of morphological and immunohistochemical findings with the results of mutational analyses.

## Supplementary information

Below is the link to the electronic supplementary material.

Supplementary file1 (PDF 136 KB)

## Data Availability

Data generated or analyzed during this study are included in this published article.

## References

[1] Khoury JD, Solary E, Abla O, Akkari Y, Alaggio R, Apperley JF, Bejar R, Berti E, Busque L, Chan JKC, Chen W, Chen X, Chng WJ, Choi JK, Colmenero I, Coupland SE, Cross NCP, De Jong D, Elghetany MT, Takahashi E, Emile JF, Ferry J, Fogelstrand L, Fontenay M, Germing U, Gujral S, Haferlach T, Harrison C, Hodge JC, Hu S, Jansen JH, Kanagal-Shamanna R, Kantarjian HM, Kratz CP, Li XQ, Lim MS, Loeb K, Loghavi S, Marcogliese A, Meshinchi S, Michaels P, Naresh KN, Natkunam Y, Nejati R, Ott G, Padron E, Patel KP, Patkar N, Picarsic J, Platzbecker U, Roberts I, Schuh A, Sewell W, Siebert R, Tembhare P, Tyner J, Verstovsek S, Wang W, Wood B, Xiao W, Yeung C, Hochhaus A (2022) The 5th edition of the World Health Organization classification of haematolymphoid tumours: myeloid and histiocytic/dendritic neoplasms. Leukemia 36:1703–1719. 10.1038/s41375-022-01613-1 35732831 10.1038/s41375-022-01613-1 PMC9252913

[2] Greiner G, Gurbisz M, Ratzinger F, Witzeneder N, Class SV, Eisenwort G, Simonitsch-Klupp I, Esterbauer H, Mayerhofer M, Mullauer L, Sperr WR, Valent P, Hoermann G (2020) Molecular quantification of tissue disease burden is a new biomarker and independent predictor of survival in mastocytosis. Haematologica 105:366–374. 10.3324/haematol.2019.217950 31018976 10.3324/haematol.2019.217950 PMC7012478

[3] Bartels S, Hasemeier B, Vogtmann J, Schipper E, Busche G, Schlue J, Kreipe H, Lehmann U (2020) Feasibility of combined detection of gene mutations and fusion transcripts in bone marrow trephines from leukemic neoplasms. J Mol Diagn 22:591–598. 10.1016/j.jmoldx.2020.01.004 32036087 10.1016/j.jmoldx.2020.01.004

[4] Bartels S, Lehmann U (2015) Analysis of mutational hotspots in routinely processed bone marrow trephines by pyrosequencing(®). Methods Mol Biol 1315:103–114. 10.1007/978-1-4939-2715-9_8 26103894 10.1007/978-1-4939-2715-9_8

[5] Erben P, Schwaab J, Metzgeroth G, Horny HP, Jawhar M, Sotlar K, Fabarius A, Teichmann M, Schneider S, Ernst T, Muller MC, Giehl M, Marx A, Hartmann K, Hochhaus A, Hofmann WK, Cross NC, Reiter A (2014) The KIT D816V expressed allele burden for diagnosis and disease monitoring of systemic mastocytosis. Ann Hematol 93:81–88. 10.1007/s00277-013-1964-1 24281161 10.1007/s00277-013-1964-1

[6] Agarwal R, McBean M, Hewitt C, Westerman DA (2014) KIT D816V mutation detection: a comparative study using peripheral blood, bone marrow aspirate and bone marrow trephine samples for detection of KIT mutations in patients with systemic mastocytosis. Leuk Lymphoma 55:2202–2203. 10.3109/10428194.2013.876498 24359244 10.3109/10428194.2013.876498

[7] Tan A, Westerman D, McArthur GA, Lynch K, Waring P, Dobrovic A (2006) Sensitive detection of KIT D816V in patients with mastocytosis. Clin Chem 52:2250–2257. 10.1373/clinchem.2006.068205 17040960 10.1373/clinchem.2006.068205

[8] Kähler C, Didlaukat S, Feller AC, Merz H (2007) Sensitive and reliable detection of Kit point mutation Asp 816 to Val in pathological material. Diagn Pathol 2:37. 10.1186/1746-1596-2-37 17900365 10.1186/1746-1596-2-37 PMC2211455

[9] Schumacher JA, Elenitoba-Johnson KS, Lim MS (2008) Detection of the c-kit D816V mutation in systemic mastocytosis by allele-specific PCR. J Clin Pathol 61:109–114. 10.1136/jcp.2007.047928 17526803 10.1136/jcp.2007.047928

[10] Kristensen T, Vestergaard H, Moller MB (2011) Improved detection of the KIT D816V mutation in patients with systemic mastocytosis using a quantitative and highly sensitive real-time qPCR assay. J Mol Diagn 13:180–188. 10.1016/j.jmoldx.2010.10.004 21354053 10.1016/j.jmoldx.2010.10.004 PMC3279709

[11] Sotlar K, Escribano L, Landt O, Mohrle S, Herrero S, Torrelo A, Lass U, Horny HP, Bultmann B (2003) One-step detection of c-kit point mutations using peptide nucleic acid-mediated polymerase chain reaction clamping and hybridization probes. Am J Pathol 162:737–746. 10.1016/S0002-9440(10)63870-9 12598308 10.1016/S0002-9440(10)63870-9 PMC1868096

[12] Corless CL, Harrell P, Lacouture M, Bainbridge T, Le C, Gatter K, White C Jr, Granter S, Heinrich MC (2006) Allele-specific polymerase chain reaction for the imatinib-resistant KIT D816V and D816F mutations in mastocytosis and acute myelogenous leukemia. J Mol Diagn 8:604 17065430 10.2353/jmoldx.2006.060089 PMC1876167

[13] Kristensen T, Broesby-Olsen S, Vestergaard H, Bindslev-Jensen C, Moller MB, Mastocytosis Centre Odense University H (2017) Comparison of gDNA-based versus mRNA-based KIT D816V mutation analysis reveals large differences between blood and bone marrow in systemic mastocytosis. Br J Haematol 178:330–332. 10.1111/bjh.14123 27196380 10.1111/bjh.14123

[14] Kristensen T, Broesby-Olsen S, Vestergaard H, Bindslev-Jensen C, Moller MB, Mastocytosis Centre Odense University H (2016) Targeted ultradeep next-generation sequencing as a method for KIT D816V mutation analysis in mastocytosis. Eur J Haematol 96:381–388. 10.1111/ejh.12601 26095448 10.1111/ejh.12601

