# Supplementary material for: Sensitive and reliable detection of KIT p.D816V mutation in decalcified archival bone marrow trephines
Source: Virchows Arch. 2024 Nov 14;486(6):1349–53. doi: 10.1007/s00428-024-03973-8 (PMC12213911; doi:10.1007/s00428-024-03973-8)
Supplement: Supplementary file 1 — Supplementary file1 (PDF 136 KB) [file 428_2024_3973_MOESM1_ESM.pdf]

Supplementary Information

Materials and Methods supplements

Tissue processing
Bone marrow trephines were fixed for at least 24 hours in bone marrow fixative [64% methanol (v/v) (J.T. Baker, Hampton, NH), 4.48 mol/L formaldehyde (Merck, Darmstadt, Germany), 1.6 mmol/L sodium hydrogen phosphate pH 7.4, 0.0074 mol/L glucose]. Fixed trephines were decalcified in the ultrasonic decalcifying automat USE 33 (Medite, Burgdorf, Germany) by incubation at 16°C overnight in EDTA solution [0.0272 mol/L Tris (Merck) and 0.270 mol/L EDTA (Merck), pH 7.4]. After washing in water trephines were embedded in paraffin.

DNA and RNA extraction
DNA and RNA isolation were performed from seven to ten whole slides (10-µm thick) depending from the trephine size using the Maxwell RSC instrument and reagents recommended by the manufacturer for FFPE material (Promega, Madison, WI). DNA concentrations were measured with a Qubit 2.0 fluorometer instrument (Invitrogen, Darmstadt, Germany) using double-stranded DNA high-sensitivity kit (Life Technologies, Carlsbad, CA) and for RNA concentrations the NanoDrop 2000 (ThermoFisher Scientific, Waltham, MA).

cDNA synthesis
cDNA synthesis were performed with the „High Capacity cDNA Reverse Transcriptase-KIT“ (Thermo Fisher Scientific, Waltham, MA, USA) according to the manufacturer`s instructions with 200 – 1000 ng of total RNA depending of the amount of isolated RNA. In the majority of the specimens we could use 800 – 1000 ng, but there were some cases with low RNA concentrations in which we only could use up to 10 µL RNA solution. The kit contains random RT-primers.

Pyrosequencing
For one preparation 15 ng gDNA or 3 µL cDNA from a total volume of 20 µL cDNA sythesis reaction volume were used. The gDNA or cDNA were amplified by PCR separated for the forward and the reverse strands with biotinylated primers:
Forward assay: Forward: 5’-GCA GCC AGA AAT ATC CTC CTT ACT CAT-3’
Reverse: 5’-Biotin-CCA CAT AAT TAG AAT CAT TCT TGA-3’

Reverse assay: Forward: 5’-Biotin-AGA TTT GTG ATT TTG GTC TAG CC-3’
Reverse: 5’-CGT TTC CTT TAA CCA CAT AAT TAG A-3’

The pyrosequencing was done with PyromarkQ24 (Qiagen, Hilden, Germany) and the PyroMarkQ24 station with the VacuumPrepTool (Qiagen, Hilden, Germany) and streptavidin sepharose beads and buffers according to the manufacturer’s instructions. The following primers were used for the pyrosequencing:
Forward 5‘-TGT GAT TTT GGT CTA GC-3‘
Reverse 5‘-TTA GAA TCA TTC TTG ATG-3‘

DigitalPCR
The primer sequences of the assay “KIT_1314” (Assay ID:Hs000000039_rm, Thermo Fisher Scientific) were not published, but the context sequence was showed off by the manufacturer : ATTTGTGATTTTGGTCTAGCCAGAG[A/T]CATCAAGAATGATTCTAATTATGTG.

Technical sensitivities

The following three figures S1, S2 and S3 show the results of the dilution series in all three assays.

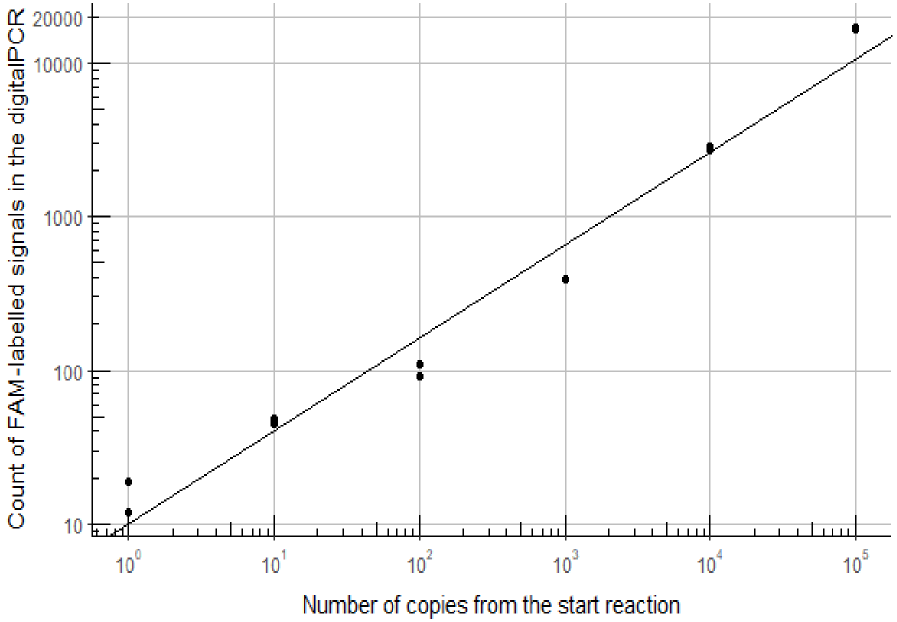

Supplemental Figure S1

Figure S1 illustrates the dependence of the count of FAM-labelled signals on used oligonucleotide as template in die digitalPCR in a twofold design.

a) b)

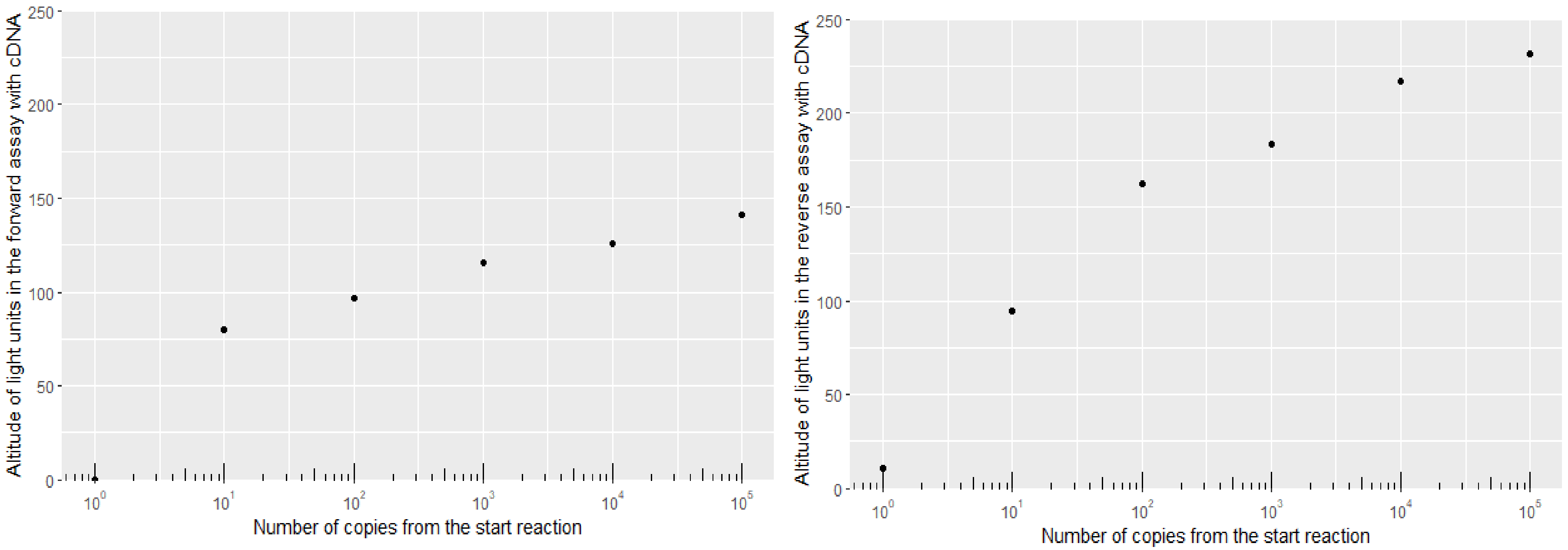

Supplemental Figure S2

Figure S2 shows the dependencies of the altitude of the light units in the pyrosequencing reaction on the used plasmide with cDNA insert as template. The results are presented separately for the forward (a) and the reverse assay (b).

a)

b)

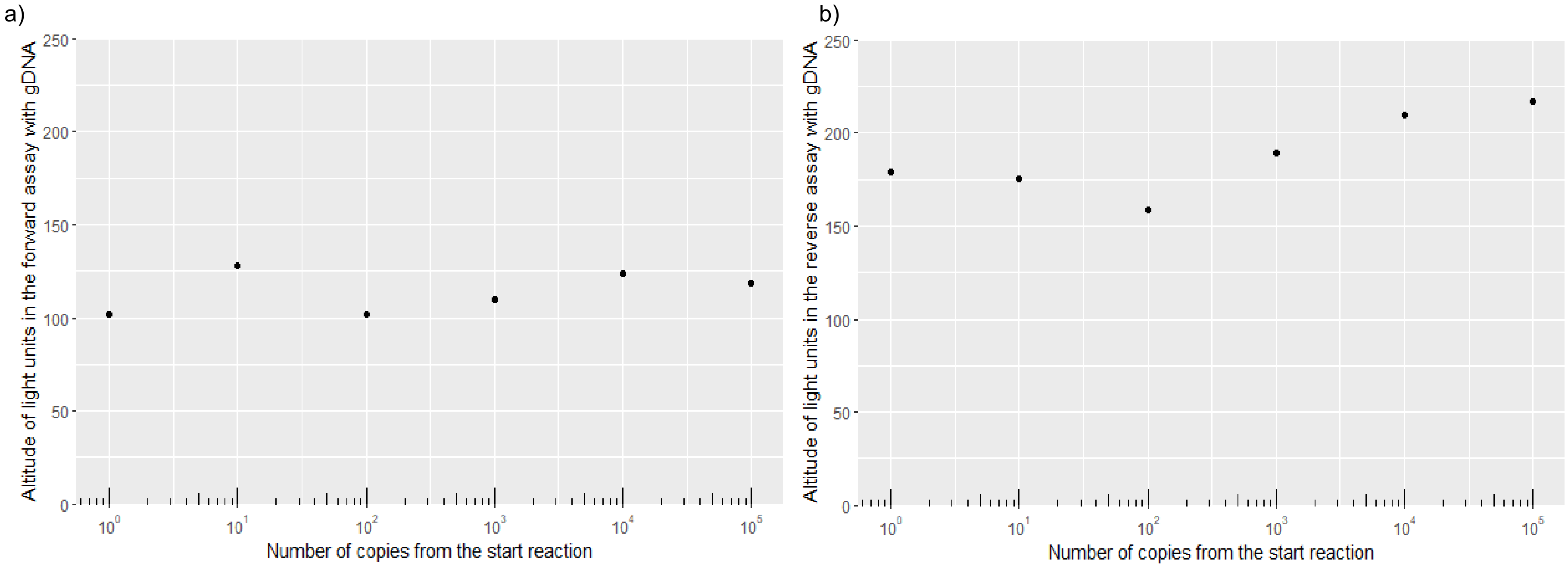

Supplemental Figure S3

Figure S3 shows the dependencies of the altitude of the light units in the pyrosequencing reaction on the used oligonucleotide as template. The results are presented separately for the forward (a) and the reverse assay (b).

Supplemental table S1: Sample data

ID	Diagnosis	AHN or other neoplasia	Tissue	Age	dPCR results	Avg. FAM signals absolute	Avg. dPCR VAF in %
1	SM	none	bone marrow	30	c.2447A>T	52.5	1.50
2	SM	none	bone marrow	49	c.2447A>T	160.5	3.15
3	SM	none	bone marrow	60	c.2447A>T	240.0	5.41
4	SM	none	bone marrow	64	c.2447A>T	31.5	0.64
5	SM	none	bone marrow	46	c.2447A>T	165.0	3.61
6	SM	none	bone marrow	57	c.2447A>T	11.5	0.35
7	SM	none	bone marrow	60	c.2447A>T	60.5	1.29
8	SM	none	bone marrow	64	c.2447A>T	193.0	3.20
9	SM	none	bone marrow	69	c.2447A>T	271.0	5.56
10	SM	none	bone marrow	65	c.2447A>T	17.5	0.38
11	SM	none	bone marrow	73	c.2447A>T	135.0	2.61
12	SM	none	bone marrow	63	c.2447A>T	262.5	4.76
13	SM	none	bone marrow	39	c.2447A>T	167.0	3.62
14	SM	none	bone marrow	27	c.2447A>T	83.0	1.90
15	SM	none	bone marrow	82	c.2447A>T	519.0	10.16
16	SM	none	bone marrow	67	c.2447A>T	119.5	2.46
17	SM	none	bone marrow	56	c.2447A>T	93.5	1.88
18	SM	none	bone marrow	32	c.2447A>T	37.0	0.85
19	SM	none	bone marrow	78	c.2447A>T	5.5	0.10
20	SM	none	bone marrow	62	c.2447A>T	20.0	1.10
21	SM	none	bone marrow	54	c.2447A>T	51.0	0.99
22	SM	none	bone marrow	66	c.2447A>T	37.0	0.85
23	SM	none	bone marrow	25	c.2447A>T	31.0	2.72
24	SM	none	bone marrow	71	c.2447A>T	139.0	3.24
25	SM	none	bone marrow	61	c.2447A>T	19.0	0.68
26	SM	MGUS	bone marrow	53	c.2447A>T	643.5	15.28
27	SM	none	bone marrow	62	c.2447A>T	467.0	12.19
28	SM	none	bone marrow	42	c.2447A>T	56.5	2.13
29	SM	CHIP	bone marrow	44	c.2447A>T	139.5	3.74
30	SM	none	bone marrow	57	c.2447A>T	49.5	1.19
31	SM	none	bone marrow	65	c.2447A>T	306.0	9.02

ID	Diagnosis	AHN or other neoplasia	Tissue	Age	dPCR results	Avg. FAM signals absolute	Avg. dPCR VAF in %
32	SM	none	colon	58	c.2447A>T	72.0	5.52
33	SMAHN	CMML-0	bone marrow	74	c.2447A>T	2438.5	37.38
34	SMAHN	CEL	bone marrow	69	c.2447A>T	877.5	26.56
35	SMAHN	MDS/MPN-U	bone marrow	58	c.2447A>T	3696.0	45.64
36	SMAHN	CMML-1	bone marrow	64	c.2447A>T	4047.0	47.98
37	SMAHN	AML in remission	bone marrow	76	c.2447A>T	95.5	2.34
38	SMAHN	Plasmocytoma	bone marrow	59	c.2447A>T	3239.5	41.20
39	SMAHN	CMML-0	bone marrow	75	c.2447A>T	3038.0	44.72
40	SMAHN	AML in remission	bone marrow	65	c.2447A>T	50.5	1.11
41	SMAHN	CEL	bone marrow	58	c.2447A>T	5.0	0.20
42	SMAHN	CEL	bone marrow	61	wt	2.0	0.11
43	SMAHN	CMML-1	bone marrow	74	c.2447A>T	3161.5	44.75
44	SMAHN	CMML-2	bone marrow	83	c.2447A>T	2993.0	39.80
45	SMAHN	MDS with 5q deletion	bone marrow	66	c.2447A>T	9.5	0.31
46	SMAHN	PMF	bone marrow	69	c.2447A>T	22.5	0.60
47	SMAHN	MDS-EB-2 or MDS/MPN-U	bone marrow	63	c.2447A>T	59.5	1.32
48	SMAHN	Polycythemia vera	bone marrow	68	c.2447A>T	629.0	11.96
49	SMAHN	MDS-EB-1	bone marrow	67	c.2447A>T	1528.5	24.76
50	SMAHN	CMML-1 and B-CLL	bone marrow	71	c.2447A>T	3193.5	42.35
51	SMAHN	MDS-EB-1 or CMML-1	bone marrow	67	c.2447A>T	1958.5	29.00
52	SMAHN	Prefibrotic PMF	bone marrow	59	c.2447A>T	14.5	0.37
53	SMAHN	PMF and BCR-ABL subclone	bone marrow	60	c.2447A>T	6.5	0.53
54	SMAHN	MDS/MPN-U	bone marrow	56	wt	0.5	0.05
55	SMAHN	MDS-EB-1	bone marrow	61	c.2447A>T	444.0	9.91
56	SMAHN	CMML-2	bone marrow	72	c.2447A>T	2043.5	36.97
57	SMAHN	Prefibrotic PMF	bone marrow	46	wt	0.5	0.05
58	SMAHN	CML	bone marrow	74	c.2447A>T	26.0	0.99
59	SMAHN	CMML-0	bone marrow	66	c.2447A>T	364.5	8.86
60	SMAHN	MDS/MPN-U	bone marrow	78	c.2447A>T	3691.0	48.16
61	SMAHN	AML in remission	bone marrow	70	c.2447A>T	7.0	0.26
62	SMAHN	MDS in transformation in AML	bone marrow	77	c.2447A>T	10.0	0.85

ID	Diagnosis	AHN or other neoplasia	Tissue	Age	dPCR results	Avg. FAM signals absolute	Avg. dPCR VAF in %
63	SMAHN	MPN-U	bone marrow	54	c.2447A>T	10.0	0.34
64	SMAHN	Plasmocytoma	bone marrow	54	c.2447A>T	39.0	1.25
65	SMAHN	CMML-0	bone marrow	72	c.2447A>T	7.0	0.28
66	SMAHN	MPN-U	bone marrow	76	c.2447A>T	16.5	0.61
67	SMAHN	CMML-0	bone marrow	83	c.2447A>T	39.0	1.37
68	SMAHN	Plasmocytoma	bone marrow	58	wt	4.0	0.29
69	SMAHN	AML in remission	bone marrow	82	wt	0.5	0.16
70	SMAHN	CEL	bone marrow	20	wt	1.5	0.11
71	SMAHN	CMML-1	bone marrow	60	c.2447A>T	3448.0	45.53
72	SMAHN	AML	bone marrow	73	c.2447A>T	514.0	11.55
73	SMAHN	MDS/MPN-U	bone marrow	58	c.2447A>T	2587.0	36.19
74	SMAHN	B-CLL	bone marrow	65	c.2447A>T	18.5	0.65
75	MCL	none	bone marrow	21	wt	2.0	0.06
76	MCL	CMML in transformation to AML	bone marrow	82	c.2447A>T	13.0	0.32
77	*	MPN-U	bone marrow	49	wt	3.0	0.12
78	*	MDS-MLD	bone marrow	76	wt	2.0	0.23
79	*	none	bone marrow	48	wt	2.0	0.37
80	*	none	bone marrow	41	c.2447A>T	5.5	0.11
81	*	MDS/MPN-RS-T	bone marrow	76	wt	1.5	0.12
82	*	none	bone marrow	29	c.2447A>T	10.5	0.32
83	*	none	bone marrow	23	c.2447A>T	9.5	0.25
84	*	none	bone marrow	59	wt	2.5	0.19
85	*	none	bone marrow	43	wt	1.0	0.15
86	*	none	bone marrow	55	wt	1.0	0.13
87	*	none	bone marrow	65	wt	3.0	0.13
88	*	PMF	bone marrow	75	wt	1.0	0.16
89	*	none	bone marrow	70	wt	4.5	0.21
90	*	MDS-EB-1	bone marrow	53	wt	1.5	0.13
91	*	none	bone marrow	64	wt	0.0	0.12
92	*	CML	bone marrow	66	wt	0.5	0.04
93	*	CMML-1	bone marrow	76	wt	1.0	0.11
94	*	none	bone marrow	54	c.2447A>T	7.0	0.98

ID	Diagnosis	AHN or other neoplasia	Tissue	Age	dPCR results	Avg. FAM signals absolute	Avg. dPCR VAF in %
95	*	MDS-MLD	bone marrow	82	wt	4.0	0.28
96	*	none	bone marrow	59	wt	4.5	0.22
97	*	MDS-RS-SLD	bone marrow	70	wt	4.0	0.12
98	*	MDS-U and T-NHL	bone marrow	70	wt	1.5	0.10
99	*	none	bone marrow	44	wt	4.0	0.06
100	*	none	bone marrow	44	wt	0.5	0.07
101	*	none	colon	40	wt	2.5	0.17

ID	Pyro cDNA results	Avg. Pyro cDNA position 2447 EAB in %	Pyro gDNA results	Avg. Pyro gDNA position 2447 VAF in %
1	c.2447A>T	60.0	wt	2.0
2	c.2447A>T	44.5	c.2447A>T	3.0
3	c.2447A>T	60.5	c.2447A>T	4.0
4	c.2447A>T	30.5	wt	2.5
5	c.2447A>T	45.5	c.2447A>T	3.5
6	c.2447A>T	21.5	wt	1.5
7	c.2447A>T	38.0	wt	2.0
8	c.2447A>T	43.5	c.2447A>T	3.5
9	c.2447A>T	46.0	c.2447A>T	3.0
10	c.2447A>T	26.0	wt	1.5
11	c.2447A>T	32.5	wt	2.5
12	c.2447A>T	43.5	c.2447A>T, c.2443A>G	4.5
13	c.2447A>T	40.5	c.2447A_T	3.5
14	c.2447A>T	37.0	wt	2.0
15	c.2447A>T	38.5	c.2447A>T	7.5
16	c.2447A>T	40.0	c.2447A>T	3.0
17	c.2447A>T	45.0	wt	2.0
18	c.2447A>T	32.0	wt	2.0
19	c.2447A>T	4.0	wt	1.0
20	c.2447A>T	46.0	wt	1.5
21	c.2447A>T	40.0	wt	2.0

ID	Pyro cDNA results	Avg. Pyro cDNA position 2447 EAB in %	Pyro gDNA results	Avg. Pyro gDNA position 2447 VAF in %
22	c.2447A>T	27.0	wt	2.0
23	c.2447A>T	44.5	wt	2.0
24	c.2447A>T	35.0	c.2447A>T	3.0
25	c.2447A>T	19.0	wt	1.5
26	c.2447A>T	43.0	c.2447A>T	10.0
27	c.2447A>T	38.5	c.2447A>T	8.0
28	c.2447A>T, c.2446G>C	28.0	wt	2.0
29	c.2447A>T	32.5	c.2447A>T	3.0
30	c.2447A>T	27.0	wt	1.5
31	c.2447A>T	42.0	c.2447A>T	6.0
32	c.2447A>T	27.5	c.2447A>T	3.0
33	c.2447A>T	31.5	c.2447A>T	33.5
34	c.2447A>T	43.0	c.2447A>T	16.5
35	c.2447A>T	48.5	c.2447A>T	40.5
36	c.2447A>T	47.0	c.2447A>T	42.0
37	c.2447A>T	39.5	wt	2.0
38	c.2447A>T	46.0	c.2447A>T	37.5
39	c.2447A>T	45.0	c.2447A>T	40.5
40	c.2447A>T	40.0	wt	2.0
41	wt	2.0	wt	1.0
42	c.2446G>C	2.0	wt	1.0
43	c.2447A>T	47.5	c.2447A>T	42.0
44	c.2447A>T	43.5	c.2447A>T	34.0
45	c.2447A>T	5.0	wt	1.0
46	wt	2.5	wt	2.0
47	wt	1.5	wt	2.5
48	c.2447A>T	42.5	c.2447A>T	9.5
49	c.2447A>T	38.0	c.2447A>T	20.5
50	c.2447A>T	47.5	c.2447A>T	37.5
51	c.2447A>T	39.5	c.2447A>T	24.5

ID	Pyro cDNA results	Avg. Pyro cDNA position 2447 EAB in %	Pyro gDNA results	Avg. Pyro gDNA position 2447 VAF in %
52	wt	1.5	wt	1.5
53	wt	1.0	wt	1.0
54	wt	1.5	wt	1.0
55	c.2447A>T, c.2446G>C	13.5	c.2447A>T	7.0
56	c.2447A>T	37.5	c.2447A>T	30.0
57	c.2447A>T	36.5	wt	1.5
58	c.2447A>T	31.0	wt	1.5
59	c.2447A>T	32.5	c.2447A>T	7.5
60	c.2447A>T	46.0	c.2447A>T	44.5
61	wt	1.5	c.2447A>T	4.5
62	wt	1.5	wt	1.5
63	c.2447A>T	8.5	wt	1.0
64	c.2447A>T	26.5	wt	1.5
65	wt	1.0	wt	1.0
66	c.2447A>T	30.0	wt	1.0
67	c.2447A>T	37.5	wt	2.0
68	c.2447A>T	7.5	wt	1.0
69	c.2446G>C	2.0	wt	1.0
70	c.2446G>C	3.0	wt	1.0
71	c.2447A>T	46.0	c.2447A>T	40.5
72	c.2447A>T	30.5	c.2447A>T	9.5
73	c.2447A>T	41.5	c.2447A>T	31.0
74	c.2447A>T, c.2446G>C	13.5	wt	1.0
75	wt	2.0	wt	1.0
76	c.2446G>C	1.0	c.2446G>C	1.0
77	c.2446G>C	1.0	wt	1.0
78	wt	1.5	wt	1.5
79	wt	1.0	wt	1.0
80	wt	1.0	wt	1.0
81	wt	2.0	wt	1.0

ID	Pyro cDNA results	Avg. Pyro cDNA position 2447 EAB in %	Pyro gDNA results	Avg. Pyro gDNA position 2447 VAF in %
82	wt	1.5	wt	1.0
83	wt	1.0	wt	1.0
84	wt	1.5	wt	1.0
85	wt	1.5	wt	1.0
86	wt	1.5	wt	1.5
87	wt	3.5	wt	1.0
88	wt	1.0	wt	1.0
89	wt	3.0	wt	1.0
90	wt	1.5	wt	1.0
91	wt	1.5	wt	1.5
92	wt	1.0	wt	1.0
93	wt	1.0	wt	1.0
94	c.2447A>T	38.5	wt	1.0
95	wt	1.0	wt	1.0
96	c.2447A>T	16.0	wt	1.0
97	wt	1.0	wt	2.0
98	wt	1.5	wt	1.0
99	wt	1.0	wt	1.0
100	wt	1.5	wt	1.0
101	wt	2.5	wt	1.0

ID	Mast cell count in % of all cells	Spindle shaped mast cells in %	Hypogranulation of mast cells in %	Count of mast cells in compact infiltrates
1	20	80	20	≥ 15
2	15	90	90	≥ 15
3	10	95	80	≥ 15
4	1	70	70	5
5	30	80	90	≥ 15
6	5	70	90	≥ 15
7	8	90	100	12
8	5	80	30	10
9	8	90	90	10
10	3	90	60	≥ 15
11	5	40	0	5
12	30	10	80	≥ 15
13	20	80	50	≥ 15
14	15	70	20	≥ 15
15	8	60	60	≥ 15
16	5	70	30	≥ 15
17	8	70	70	≥ 15
18	3	50	60	6
19	2	75	75	≥ 15
20	3	90	50	0
21	8	80	80	≥ 15
22	2	30	10	0
23	5	10	30	≥ 15
24	15	80	10	0
25	5	75	80	≥ 15
26	7	60	50	≥ 15
27	10	60	80	3
28	7	60	10	≥ 15
29	15	40	20	≥ 15
30	15	30	10	≥ 15
31	35	50	10	≥ 15

ID	Mast cell count in % of all cells	Spindle shaped mast cells in %	Hypogranulation of mast cells in %	Count of mast cells in compact infiltrates
32	20	40	80	≥ 15
33	30	70	90	≥ 15
34	3	80	80	≥ 15
35	20	70	90	≥ 15
36	15	90	90	≥ 15
37	5	80	80	5
38	55	80	50	≥ 15
39	40	80	90	≥ 15
40	5	90	90	≥ 15
41	5	80	80	3
42	30	20	90	≥ 15
43	7	90	80	≥ 15
44	12	80	90	≥ 15
45	8	10	50	0
46	8	80	20	5
47	10	80	30	12
48	15	15	85	≥ 15
49	40	80	80	≥ 15
50	35	80	40	≥ 15
51	10	20	70	≥ 15
52	3	35	10	0
53	10	95	90	0
54	50	0	60	≥ 15
55	5	30	10	0
56	5	30	30	5
57	7	40	60	≥ 15
58	5	15	40	≥ 15
59	10	50	70	≥ 15
60	5	70	70	≥ 15
61	80	20	90	≥ 15

ID	Mast cell count in % of all cells	Spindle shaped mast cells in %	Hypogranulation of mast cells in %	Count of mast cells in compact infiltrates
62	30	40	50	≥ 15
63	2	60	10	0
64	7	80	20	≥ 15
65	7	90	10	≥ 15
66	5	80	80	≥ 15
67	6	80	50	5
68	3	70	70	5
69	70	70	50	5
70	4	50	20	0
71	70	20	75	≥ 15
72	20	50	80	≥ 15
73	50	35	90	≥ 15
74	4	80	80	10
75	85	0	0	0
76	65	5	5	≥ 15
77	2	20	0	0
78	10	75	50	3
79	0.5	5	0	0
80	0.5	10	0	0
81	2	35	35	0
82	3	0	0	0
83	0.5	0	0	0
84	1	0	0	0
85	10	20	20	≥ 15
86	8	20	20	0
87	0.5	0	0	0
88	3	20	20	0
89	0	0	0	0
90	2	50	50	0
91	2	2	0	0

ID	Mast cell count in % of all cells	Spindle shaped mast cells in %	Hypogranulation of mast cells in %	Count of mast cells in compact infiltrates
92	7	5	0	0
93	7	70	80	0
94	10	15	0	0
95	10	70	70	5
96	1	5	0	0
97	4	80	10	3
98	15	90	50	0
99	20	20	0	2
100	7	10	0	2
101	5	5	0	0

ID	CD25 expression	Serum tryptase levels in µg/L
1	positive	not available
2	positive	not available
3	positive	72.0
4	positive	not available
5	positive	not available
6	positive	not available
7	positive	not available
8	positive	28.3
9	positive	not available
10	positive	14.6
11	positive	not available
12	positive	not available
13	positive	not available
14	positive	19.7
15	positive	not available
16	positive	46.6
17	positive	not available
18	positive	27.0
19	positive	not available
20	positive	not available
21	positive	not available
22	positive	not available
23	positive	not available
24	positive	48.8
25	negative	not available
26	positive	not available
27	positive	not available
28	positive	not available
29	positive	not available
30	positive	not available
31	positive	169.0

ID	CD25 expression	Serum tryptase levels in µg/L
32	positive	not available
33	positive	not available
34	positive	not available
35	positive	not available
36	positive	not available
37	positive	not available
38	positive	not available
39	positive	not available
40	positive	not available
41	positive	not available
42	positive	not available
43	positive	not available
44	positive	not available
45	positive	not available
46	positive	not available
47	positive	not available
48	positive	not available
49	positive	not available
50	positive	not available
51	positive	96.0
52	positive	14.7
53	positive	not available
54	positive	not available
55	positive	not available
56	positive	38.0
57	positive	not available
58	positive	42.0
59	positive	not available
60	positive	not available
61	positive	not available

ID	CD25 expression	Serum tryptase levels in µg/L
62	positive	not available
63	positive	9.0
64	positive	not available
65	positive	not available
66	positive	not available
67	positive	not available
68	positive	22.3
69	positive	not available
70	positive	25.4
71	positive	not available
72	positive	38.0
73	positive	not available
74	positive	not available
75	negative	not available
76	positive	not available
77	negative	not available
78	positive	42.4
79	negative	not available
80	negative	15.0
81	positive	not available
82	negative	not available
83	negative	not available
84	negative	not available
85	negative	not available
86	negative	not available
87	negative	not available
88	negative	not available
89	negative	not available
90	negative	not available
91	negative	not available

ID	CD25 expression	Serum tryptase levels in µg/L
92	negative	not available
93	positive	not available
94	negative	19.8
95	negative	36.0
96	negative	not available
97	negative	not available
98	negative	not available
99	positive	15.5
100	negative	5.8
101	negative	not available

Sample Data with ID, diagnosis, AHN or other neoplasia, tissue type, age in years, the results of the three methods for detection of *KIT*-mutations in exon 17 with VAF and EAB and results of the absolute quantification in the dPCR as average absolute FAM signals, mast cell count, spindle shaped mast cells, hypogranulation, count of mast cells in compact mast cell infiltrates, CD25 expression and serum tryptase levels.
SM = systemic mastocytosis, SMAHN = systemic mastocytosis with an associated hematological neoplasm, MCL = mast cell leukemia, * = criteria for the diagnosis of a mast cell neoplasm are not fulfilled, wt = wild type, avg. = average.
